# Additional Remarks on Mr. Cruikshank's Experiments on Finery Cinder and Charcoal. Communicated to the Editors of the Medical Repository of New York

**Published:** 1803-09-01

**Authors:** Joseph Priestley


### Additional Remarks *on* Mr. Cruikshank's Experiments *on* Finery Cinder *and* Charcoal. *Communicated to the Editors of the Medical Repository of New York, by* Dr. Joseph Priestley, *in a Letter dated Northumberland, November* 15, 1802.

Though I have sent you several remarks on Mr. Cruikshank's observation on my argument for the doctrine of phlogiston, from the experiment with finery cinder and charcoal; yet as he has replied, and the French chemists, the great patrons of the new theory, acquiesce in and applaud his answer, I have been led to give more attention to the subject; and this has suggested further proofs, as they appear to me, of the fallacy of his hypothesis. That the chemists in this part of the world may have an early opportunity of forming a judgement on the state of the controversy, which is certainly of considerable importance, I send you the following distinct statement of my objections to the *principle* which Mr. Cruishank has assumed which is entirely different from that of Mr. Berthollet, or that of Dr. Woodhouse, and in my opinion more exceptionable than either of them.

1. Mr. Cruikshank's hypothesis requires, that in the process of heating finery cinder and charcoal, the oxygen in the finery cinder should quit that substance, and unite with carbon in the charcoal, in order to form *fixed air*. Since, however, this fixed air is to be decomposed, the oxygen which it has got from the finery cinder must be separated from it, and enter into the same calx again. But while the heat continues the same, I deem these contrary effects to be impossible. If the degree of heat that is applied expel oxygen from the calx, it will certainly prevent its return; consequently, if fixed air could be formed, it could not be decomposed, in these circumstances.

2. If the oxygen should quit the fixed air, nothing would remain of it but carbon, as before their union; and this is a solid substance, incapable, without the aid of oxygen, of assuming the form of *air*. Whence then comes the inflammable air in this process, which so nearly resembles that from charcoal and water, that they must have the same origin? And in this case Mr. Lavoisier himself decides that whatever is *inflammable* in it must come from *water*.

3. Admitting all that Mr. Cruikshank alleges, concerning the difference in the specific gravity, and other circumstances, between the air from finery cinder and charcoal, and that from water and charcoal, it is not so great as the difference between this latter and the light inflammable air from the metals with acids or water. Different as they may be in other respects, they are all *inflammable*, from their affinity to oxygen, and their property of uniting with it in a certain degree of heat; in consequence of which they are alike the reverse of *oxyds*, and must be classed among *combustible substances*, equally with sulphur and phosphorus.

4. If the oxygen, after quitting the finery cinder, entered into it again, it would make it finery cinder as at the first, or at least in some degree. But the calx is completely revived in this process; the iron so revived being as soluble in acids as any iron whatever.

5. If the iron should be completely revived in the former part of this process, by the oxygen wholly leaving it, I maintain that it could not by any degree of heat decompose fixed air; since this cannot be effected by the heat of a burning lens of sixteen inches diameter, in the space of several hours, in only a very few ounce measures of the air. Mr. Cruikshank's process with *bladders and a gun barrel*, I have frequently used, but I never had any satisfactory result. The scale he found on the iron, I have no doubt, came from moisture in the air or the bladders.

6. Mr. Cruikshank seems to think that charcoal cannot contain any oxygen; but Mr. Tennant's fine experiment decisively proves that it does. For where are we to look for the oxygen (which we all acknowledge to be a component part of the fixed air) which is separated from the marble, but in the charcoal that is produced? And in that it makes part of a solid substance, and does not take the form of air.

7. Since oxygen and all combustible substances unite and explode, in a certain degree of heat, the oxygen expelled from the finery cinder uniting with carbon from the charcoal when red hot must enable it to burn; and therefore, in these circumstances, there ought to be an explosion, or at least a gradual combustion of them in the course of the process, as there is when oxygen is put to the same substance and heated with it afterwards.

It is now near twenty years since this new theory was advanced, and from that time to the present I have not ceased to express my opinion of its fallacy, and to give my reasons for that opinion; but I have not till very lately been able to draw much attention to the subject. Now, however, that I have succeeded in this, I flatter myself that the controversy will come to a speedy termination.

MONTHLY

